# Osteochondroma With Odd Clinical and Radiological Presentation

**DOI:** 10.7759/cureus.65048

**Published:** 2024-07-21

**Authors:** Priyank Sharma, Purvesh Bhrambhatt, Gopal Kumar, Mahesh Saini, Sumit Raj

**Affiliations:** 1 Department of Orthopaedics, Jawaharlal Nehru Medical College, Ajmer, IND

**Keywords:** knee, benign, painful, physis, tumors, osteochondroma

## Abstract

Osteochondroma is a cartilage-capped bony projection arising on the external surface of the bone, containing a marrow cavity continuous with that of the underlying bone. This benign tumor develops within the metaphysis of long bones. The growth is directed away from the growing end of long bones. We report a case of osteochondroma, also known as exostosis in a nine-year-old male child at the medial aspect of the right leg proximally. Marginal excision of the tumor was performed and sent for histopathological examination. The growth involved physis and epiphysis besides metaphysis and was directed toward the growing end of the tibia.

## Introduction

Osteochondroma, also called exostosis, is a cartilage-capped bony projection arising on the external surface of bone [1]. They are the commonest type of primary benign bone tumors comprising 20-50% of all benign tumors of bone [2]. Some common sites for exostosis are the proximal humerus, proximal tibia, and distal femur. Usually mushroom-shaped, the lesion affects the metaphysis of long bones, such as the femur and tibia [3]. It is formed as a result of a mutation in the gene-encoding exostosin 1 (EXT 1), thus supporting the theory of osteochondroma being a true neoplasm. Other causes include prior surgery and post radiation. Multiple exostosis is hereditary and also called diaphyseal aclasis. Osteochondromas are twice as common in males than in females and present mostly in the first three decades of life. Osteochondroma's form when a piece of the epiphyseal growth plate cartilage separates and pushes through the periosteal bone cuff around the growth plate [4]. These lesions are mostly asymptomatic and found incidentally, and mostly present as swelling, which is hard, immobile, and non-tender. Surgical resection is indicated for symptomatic lesions, complications, cosmetic reasons, or malignant transformation. Excision of the tumor with free margin is the treatment of choice. Local recurrence is less than 2% if complete resection is achieved. This study was conducted for the purpose of finding some odd presentations in a very commonly presenting bone tumor.

## Case presentation

**Figure 1:**
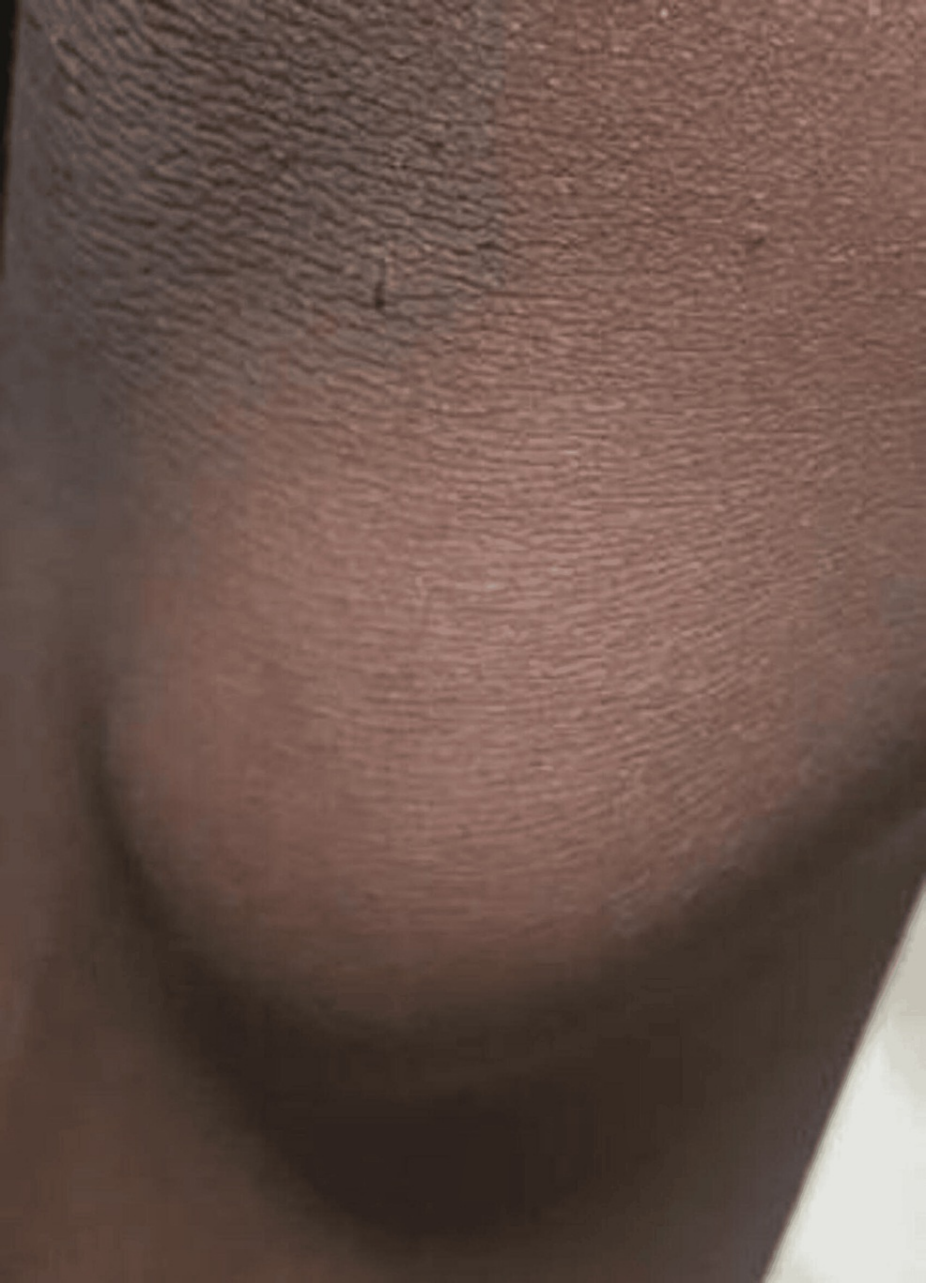
Clinical presentation of a nine-year-old male child with bony hard swelling at the medial aspect of the right proximal leg

A nine-year-old male child presented with swelling and pain over the medial aspect of the proximal leg, which first appeared when he was two years old. The swelling was insidious in onset chronically progressive in nature, associated with pain. While examining the patient, a bony hard swelling measuring 3x3 cm in dimension was noted (Figure 1). It was non-mobile and tender and was fixed to the bone but not to the skin. There was no significant limitation in movement. No significant past or family history is present. Physical examination was also not significant.

Radiological study

**Figure 2:**
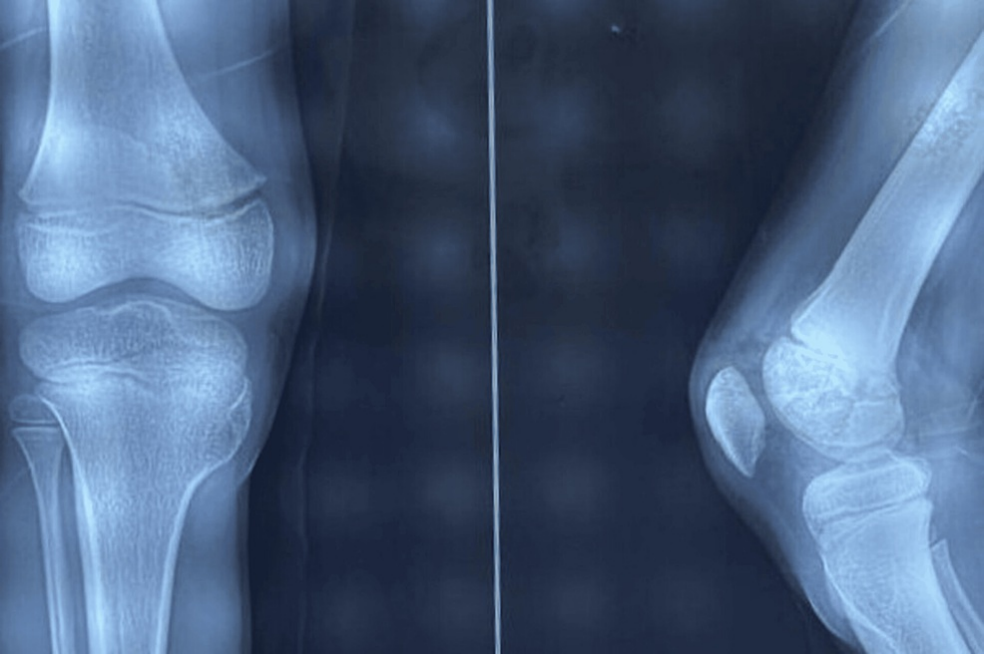
Radiograph at presentation

On anterior-posterior (AP) and lateral views of the X-ray of the right knee, there was an eccentric bony prominence noted over the medial aspect of the proximal tibia involving metaphysis, physics, and some part of epiphysis (Figure 2). The lesion was hyperdense containing a hypodense center with sharp edges. It was non-articular. No signs of fracture or dislocation are present.

The differential diagnoses looking at the radiographs include osteochondroma, pigmented villonodular synovitis, parosteal osteosarcoma, and Trevor’s disease.

**Figure 3:**
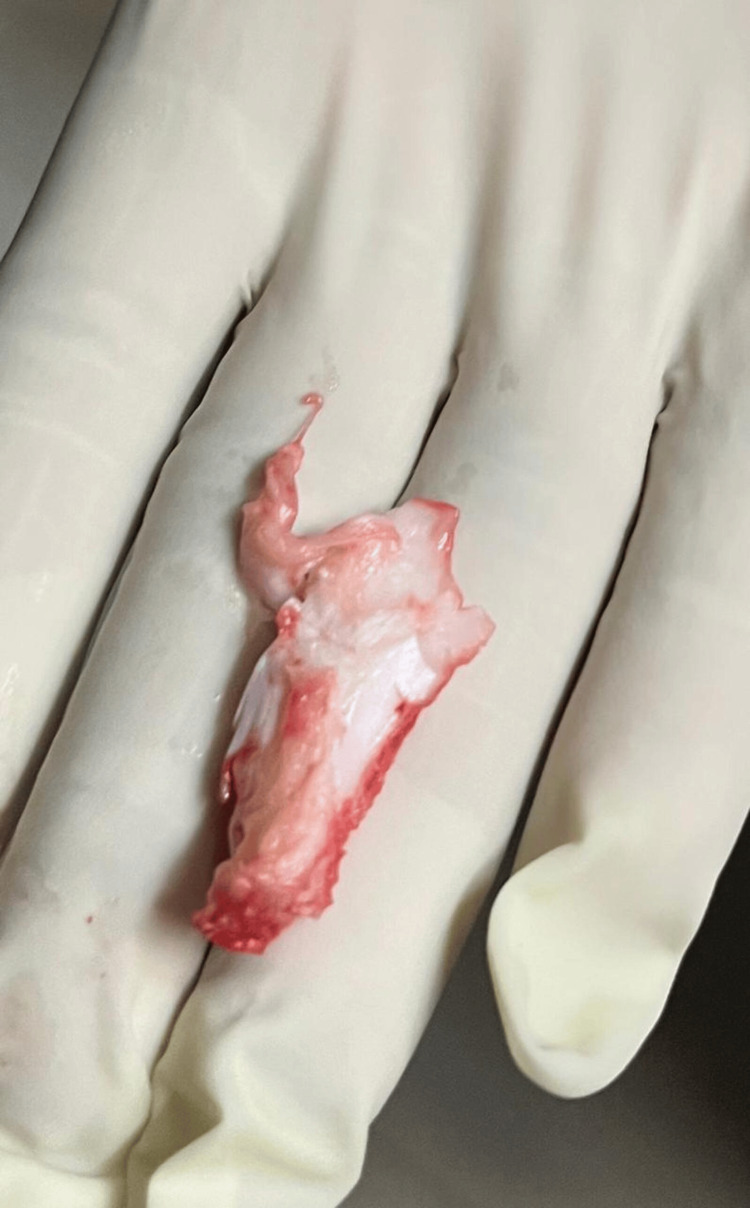
Intraoperative photograph of growth removed

**Figure 4:**
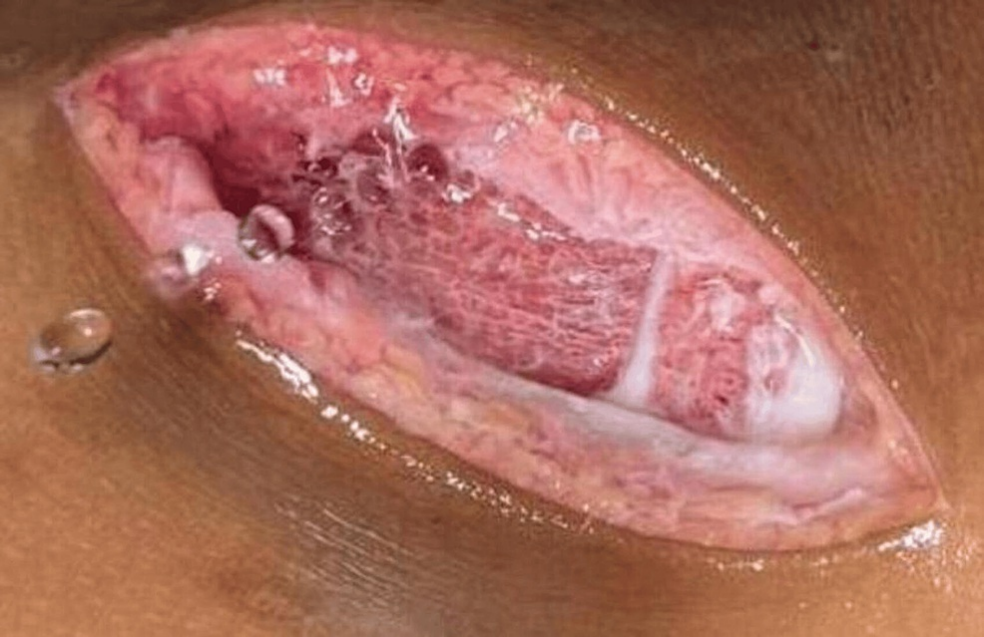
Intraoperative photograph of the surgical site showing growth involvement of epiphysis, physis, and metaphysis

The patient was planned for surgery, and a 5 cm oblique incision was made over the swelling on the medial aspect of the right proximal leg. A firm, bony, lobulated, sessile mass was seen at the medial aspect of the proximal tibia extending towards the knee joint. Cartilage was lobulated and abundant over the mass. It was resected completely (Figure 3), and the joint space was intact. After resection, it was identified that the mass involved metaphysis, physics, and some parts of epiphysis (Figure 4).

Histopathological examination

**Figure 5:**
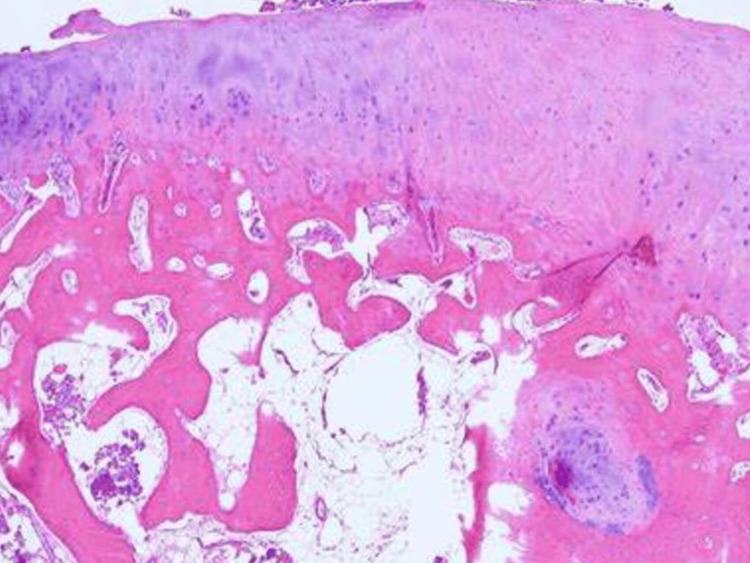
Histopathological photograph suggestive of osteochondroma

The resected tumor with margins was sent for histopathological examination. Microscopic examination reveals the outermost layer of fibrous connective tissue and a middle layer of chondrocytes arranged in clusters superficially and vertically deeper. The innermost layer consists of osteoid cells, which are trabecular and have marrow spaces intervening. These bony trabeculae are covered by a thick layer of hyaline cartilage. There is no evidence of malignancy, and features indicate it as an osteochondroma [5] (Figure 5).

The patient was discharged on the fourth postoperative day. On regular follow-ups, there were no signs and symptoms of recurrence both clinically and radiologically. The patient had a complete recovery with no limitation in range of motion and no pain.

## Discussion

Osteochondromas are a common benign bone tumor though some call these developmental malformations [6]. They are thought to originate within periosteum as small cartilaginous nodules. The growth usually ceases with skeletal maturity [6].

Additionally, 90% of patients have solitary lesions; however, multiple lesions can also be present. Clinical features include a non-tender, painless, slow-growing mass. They are usually found on the metaphysis of a long bone and are directed away from the joint. In our case, the patient was a young male child with painful swelling over the proximal leg directed toward the knee joint.

These lesions are mostly asymptomatic and discovered incidentally. Some may cause pain mainly by irritating the surrounding structures and rarely due to fracture. Other causes of pain include false aneurysms of major vessels, bursa formation [1], neuropathies, and malignant transformations. The frequency of malignant degeneration is approximately 1% for solitary type and 5-25% for hereditary multiple exostoses. Any alterations in radiological appearance, especially with ill-defined margin evolution and thickening of the cartilage cap >15 mm, are highly suggestive of chondrosarcoma [4].

Osteochondromas may be pedunculated, seen commonly with a definite stalk, or sessile with a broad base. The projectile part of the lesion has both cancellous and cortical components continuous with that of the parent bone. The lesion is covered with a cartilaginous cap often irregular and not usually seen on radiographs and requires MRI.

The radiographic presentation includes bony growth away from the joint with a cartilage cap over it and involves the metaphysis of long bones [7]. The radiographic presentation of this case is unusual due to its growth towards the joint and involvement of physis and epiphysis, in contrast to typical osteochondroma.

Thus, in accordance with the clinical, radiological, and pathological appearance, the lesion seemed to be an unusual type of osteochondroma involving epiphysis, physics, and metaphysis and directed toward the joint. It was further confirmed during surgery.

## Conclusions

Osteochondroma is a benign cartilage tumor projecting from the external surface of the bone. It is the most common benign bone tumor, is commonly seen in long bone metaphysis grows away from the physics, and is usually asymptomatic. However, in this case, we conclude that these tumors can also develop at unusual sites involving physis and epiphysis and can also be directed towards the joint, and some can be symptomatic causing pain.
